# Summary of the best evidence for dyadic disease management in patients who have undergone coronary stenting and their family caregivers

**DOI:** 10.3389/fcvm.2026.1871242

**Published:** 2026-09-23

**Authors:** Haiyan Wang, Jiaojiao Zhu, Kunning Wei, Bowen Luo, Shijia Wan, Jiaxue Wang, Shukun Yu

**Affiliations:** 1 School of Nursing, Beihua University, Jilin City, China; 2 Affiliated Hospital of Beihua University, Jilin City, China

**Keywords:** coronary stent implantation, Dyadic disease management, family caregivers, Percutaneous Coronary Intervention, self-management

## Abstract

**Objective:**

To systematically synthesize the best available evidence on dyadic disease management for patients and their family caregivers following coronary stent implantation and to develop actionable evidence-based recommendations for clinical practice.

**Methods:**

This evidence summary used the 6S evidence pyramid to guide the prioritization and retrieval of evidence sources. Chinese and international databases and relevant professional society websites were systematically searched from inception to February 28, 2026, using terms related to percutaneous coronary intervention/coronary stenting, caregivers/dyadic care, and self-management/disease management/cardiac rehabilitation. Two reviewers independently screened eligible evidence sources, assessed methodological quality using design-appropriate appraisal tools, and extracted and synthesized the evidence.

**Results:**

A total of 18 evidence sources were included, comprising 4 guidelines, 4 expert consensus statements, 2 systematic reviews, 1 evidence summary, 3 randomized controlled trials, and 4 quasi-experimental studies. Evidence synthesis yielded 27 evidence-based recommendations across five domains: assessment and planning, education and decision-making, self-management and rehabilitation, psychosocial support, and continuity of care. Key recommendations emphasized dyadic assessment of patient and caregiver needs, psychological status, caregiver burden, and readiness for collaboration; joint education and shared decision-making; caregiver-supported medication management, lifestyle modification, rehabilitation, symptom monitoring, and emergency response; psychosocial support for both members of the dyad; and coordinated discharge transition and follow-up.

**Conclusion:**

The synthesized evidence integrates previously dispersed caregiver-related findings into a structured, consent-based dyadic management pathway for post-stenting care. Clinicians should identify and assess the caregiver selected by the patient, clarify collaborative roles and goals, provide joint education and targeted skills training, and integrate caregiver support into self-management, rehabilitation, psychosocial care, discharge transition, and follow-up. This pathway offers a practical approach to incorporating caregiver participation into post-stenting secondary prevention while preserving patient autonomy and addressing the needs of both members of the dyad.

## 1 Introduction

Cardiovascular disease is the leading cause of death worldwide, accounting for approximately 20.5 million deaths annually (1). Coronary heart disease is one of the primary causes of mortality and disability (2), and percutaneous coronary intervention (PCI) is an important treatment modality, with coronary stent implantation being one of the most commonly used revascularization procedures (3, 4). However, while stent implantation can alleviate local stenosis, it cannot reverse the progression of atherosclerosis. Postoperatively, patients remain at risk for adverse outcomes such as in-stent thrombosis, recurrent cardiovascular events, and unplanned readmissions (4, 5). Therefore, standardized and continuous disease management is key to improving patient outcomes.

With the development of the “family-centered” care philosophy, increasing attention has been paid to the interdependence between patients and family caregivers in long-term cardiovascular disease management (6, 7, 10). However, current post-stent implantation management remains largely patient-centered, with emphasis on individual medication adherence, lifestyle modification, and cardiac rehabilitation (4, 8). Although relevant guidelines emphasize secondary prevention, poor adherence and difficulty maintaining long-term behavioral change remain common challenges in clinical practice (9). Meanwhile, the needs, caregiving capacity, psychological well-being, and potential roles of family caregivers are not consistently incorporated into routine post-stenting disease management.

Previous studies relevant to post-stenting care have explored various forms of caregiver involvement following PCI, including couple-based collaborative management, family-participatory early warning and symptom monitoring, family-empowerment-based cardiac rehabilitation, and spouse-involved exercise interventions (26, 27, 31, 32). However, these studies differ in their intervention focus, mode of caregiver participation, and stage of care, resulting in evidence that is distributed across different aspects of post-PCI and post-stenting management (26, 27, 31, 32). Consequently, systematic integration of these findings into practice-oriented recommendations addressing the joint assessment, support, and participation of patients and family caregivers throughout the care continuum remains limited.

Given the limited integration of existing evidence, we conducted an evidence summary of dyadic disease management for patients who have undergone coronary stent implantation and their family caregivers, with the aim of translating the best available evidence into actionable recommendations for clinical practice. This study has been registered with the Center for Evidence-Based Nursing at Fudan University (Registration No.: ES202610161).

## 2 Methods

### 2.1 Identifying evidence-based questions

This study was conducted as an evidence summary to systematically identify, appraise, synthesize, and grade the best available evidence relevant to dyadic disease management following coronary stent implantation. Evidence-based questions were formulated using the PIPOST framework (11), which was selected because its Professional, Setting, and Type of evidence components enable explicit specification of the professionals responsible for implementation, the settings in which the evidence is applied, and the range of evidence sources considered, consistent with the practice-oriented aim of this evidence summary. The evidence-based questions were defined as follows: (1) The target population (Population) consists of patients and family caregivers aged ≥18 years following coronary stent implantation; (2) The intervention (Intervention) includes post-stenting dyadic disease-management strategies involving self-management, symptom monitoring, rehabilitation, psychosocial support, and continuity of care; (3) The professionals implementing the evidence (Professional) are healthcare professionals involved in post-stenting care; (4) Outcomes include patients' self-management abilities, self-efficacy, and levels of social support, as well as caregivers’ burden and self-efficacy; (5) Settings for evidence application include hospitals, communities, or homes; and (6) Types of evidence include clinical decision aids, guidelines, expert consensus statements, systematic reviews, evidence summaries, randomized controlled trials (RCTs), and quasi-experimental studies.

### 2.2 Search resources and strategies

#### 2.2.1 Websites and databases consulted

The selection and sequencing of evidence sources were guided by the 6S evidence pyramid model (12), which organizes evidence resources into six hierarchical levels: Systems, Summaries, Synopses of Syntheses, Syntheses, Synopses of Single Studies, and Studies. Two researchers independently conducted a top-down search, prioritizing higher-level evidence resources and subsequently searching lower-level sources when necessary. Guideline websites included the UK's National Institute for Health and Care Excellence (NICE), the Registered Nurses' Association of Ontario (RNAO), the Scottish Intercollegiate Guidelines Network (SIGN), the Guidelines International Network (GIN), the Australian Clinical Practice Guidelines Portal (ACGP), the Australian National Health and Medical Research Council (NHMRC), and the ECRI Guidelines Trust. Evidence-based resources included BMJ Best Practice, UpToDate, the Cochrane Library, and the Joanna Briggs Institute (JBI) evidence resource. Bibliographic databases included PubMed, Embase, CINAHL, Web of Science, China National Knowledge Infrastructure (CNKI), Wanfang Data Knowledge Service Platform, VIP Database, and the Chinese Biomedical Literature Database (CBM). Websites of professional societies and relevant organizations included the American Heart Association (AHA), the American College of Cardiology (ACC), the American Association of Cardiovascular and Pulmonary Rehabilitation (AACVPR), the European Society of Cardiology (ESC), and the Agency for Healthcare Research and Quality (AHRQ). Supplementary searches included Chinese medical information platforms such as MedLive and MedSci, as well as relevant gray-literature sources. Searches were conducted from database inception to February 28, 2026.

#### 2.2.2 Search strategy

A combination of controlled vocabulary and free-text terms was used. English search terms included: Percutaneous Coronary Intervention, Coronary Stenting, PCI, Stents; Caregivers, Family Caregiver, Spouse, Dyadic Care; Self-Management, Patient Compliance, Cardiac Rehabilitation, Secondary Prevention, etc. Chinese search terms include: Percutaneous Coronary Intervention, PCI, Coronary Stenting, Post-Stenting; Dyadic Care, Family Caregiver, Family Member, Family Support; Self-Management, Disease Management, Adherence, Cardiac Rehabilitation, etc. Taking CNKI as an example, the Chinese search query is: SU = (“percutaneous coronary intervention” + “PCI” + “coronary artery stenting” + “post-stenting”) AND SU = (“dyadic” + “family members” + “caregivers” + “family care” + “family support”) AND SU = (“self-management” + “disease management” + “adherence” + “cardiac rehabilitation”). Taking PubMed as an example, the English search strategy is shown in Table 1.

**Table 1:** English search strategy (using pubMed as an example).

Search	Query	Items found
#1	(“Percutaneous Coronary Intervention”(MeSH) OR “Angioplasty, Balloon, Coronary”(MeSH) OR “Stents”(MeSH) OR “Coronary Artery Disease”(MeSH)) OR (“Percutaneous Coronary Intervention”(Title/Abstract) OR “PCI”(Title/Abstract) OR “Coronary Stenting”(Title/Abstract) OR “Coronary Stent Implantation”(Title/Abstract))	237,296
#2	(“Caregivers”(MeSH)) OR (“Caregiver*”(Title/Abstract) OR “Carer*”(Title/Abstract) OR “Family Caregiver*”(Title/Abstract) OR “Spouse*”(Title/Abstract) OR “Husband”(Title/Abstract) OR “Wife”(Title/Abstract) OR “Dyad*”(Title/Abstract) OR “Dyadic Care”(Title/Abstract))	217,637
#3	(“Self-Management”(MeSH) OR “Patient Compliance”(MeSH) OR “Rehabilitation”(MeSH)) OR (“Self-Management”(Title/Abstract) OR “Self-Care”(Title/Abstract) OR “Disease Management”(Title/Abstract) OR “Adherence”(Title/Abstract) OR “Lifestyle”(Title/Abstract) OR “Behavior Change”(Title/Abstract) OR “Cardiac Rehabilitation”(Title/Abstract) OR “Secondary Prevention”(Title/Abstract))	880,878
#4	#1 AND #2 AND #3	40

### 2.3 Inclusion and exclusion criteria for evidence sources

Inclusion criteria were as follows: (1) The evidence was relevant to patients following coronary stent implantation and their family caregivers; (2) The study focused on dyadic disease management for patients and family caregivers following coronary stent implantation; (3) For primary intervention studies, including randomized controlled trials (RCTs) and quasi-experimental studies, eligible interventions were those related to post-stenting self-management, adherence, cardiac rehabilitation, or other disease-management strategies with explicit patient–caregiver participation or support; (4) Eligible evidence types included clinical decision aids, guidelines, expert consensus statements, systematic reviews, evidence summaries, RCTs, and quasi-experimental studies; and (5) Publications were limited to Chinese or English.

Exclusion criteria were as follows: (1) Studies for which the full text was unavailable or information was incomplete; (2) Duplicate publications; (3) Interpretations, translations, or non-original versions of guidelines or expert consensus statements; and (4) Primary intervention studies focused exclusively on pharmacological therapy, stent or device efficacy, or procedural techniques without patient–caregiver participation or support relevant to post-stenting dyadic disease management.

### 2.4 Evaluation of literature quality

The appropriate quality appraisal tool was selected according to the type of evidence. Clinical guidelines were evaluated using the Appraisal of Guidelines for Research and Evaluation II (AGREE II) (13). For expert consensus statements, systematic reviews, evidence summaries, quasi-experimental studies, and randomized controlled trials, the corresponding Joanna Briggs Institute (JBI) critical appraisal tools (2016 edition) were used (14). These design-specific tools were used to evaluate the methodological quality of the included evidence using criteria appropriate to each evidence type. Quality appraisal was conducted independently by two researchers trained in evidence-based methodology; disagreements were resolved through discussion or consultation with a third researcher.

### 2.5 Evidence collection, synthesis, and evaluation

Two researchers independently reviewed the included literature, extracted evidence based on the PIPOST framework (11), and organized and synthesized the extracted data; a third researcher was responsible for verification and confirmation. Extracted information included the target population, relevant management or intervention content, and evidence statements or recommendations related to the evidence-based question. Any discrepancies during the extraction process were resolved through discussion or consultation with experts in the relevant field. During evidence synthesis, evidence with consistent conclusions was combined to form unified recommendations; complementary evidence was logically synthesized; evidence with discrepancies was evaluated based on methodological quality and timeliness of publication, with the most appropriate evidence selected; and relatively independent evidence was retained separately. Evidence addressing the same or related management issues was compared for content and consistency, and the synthesized recommendations were organized by management focus and linked to their supporting sources, evidence levels, and recommendation grades.

Following evidence synthesis, the 2014 JBI Levels of Evidence were used to classify the evidence supporting each recommendation into Levels 1–5 and their corresponding sublevels, with Level 1 representing the highest level of evidence and Level 5 the lowest (15). The strength of each recommendation was subsequently graded as Grade A (strong recommendation) or Grade B (weak recommendation) based on consideration of feasibility, appropriateness, meaningfulness, and effectiveness (FAME).

## 3 Results

### 3.1 Basic characteristics of the included literature

A total of 1,101 records were identified and imported into EndNote. After removing 221 duplicates, 880 records remained. Following a stepwise screening process involving the review of titles, abstracts, and full texts, 18 evidence sources were ultimately included, comprising 4 guidelines (16–19), 4 expert consensus documents (7, 20–22), 2 systematic reviews (23, 24), 1 evidence summary (25), 3 randomized controlled trials (26–28), and 4 quasi-experimental studies (29–32). The literature screening process is shown in Figure 1. The basic characteristics of the included evidence sources are presented in Table 2.

**Figure 1:**
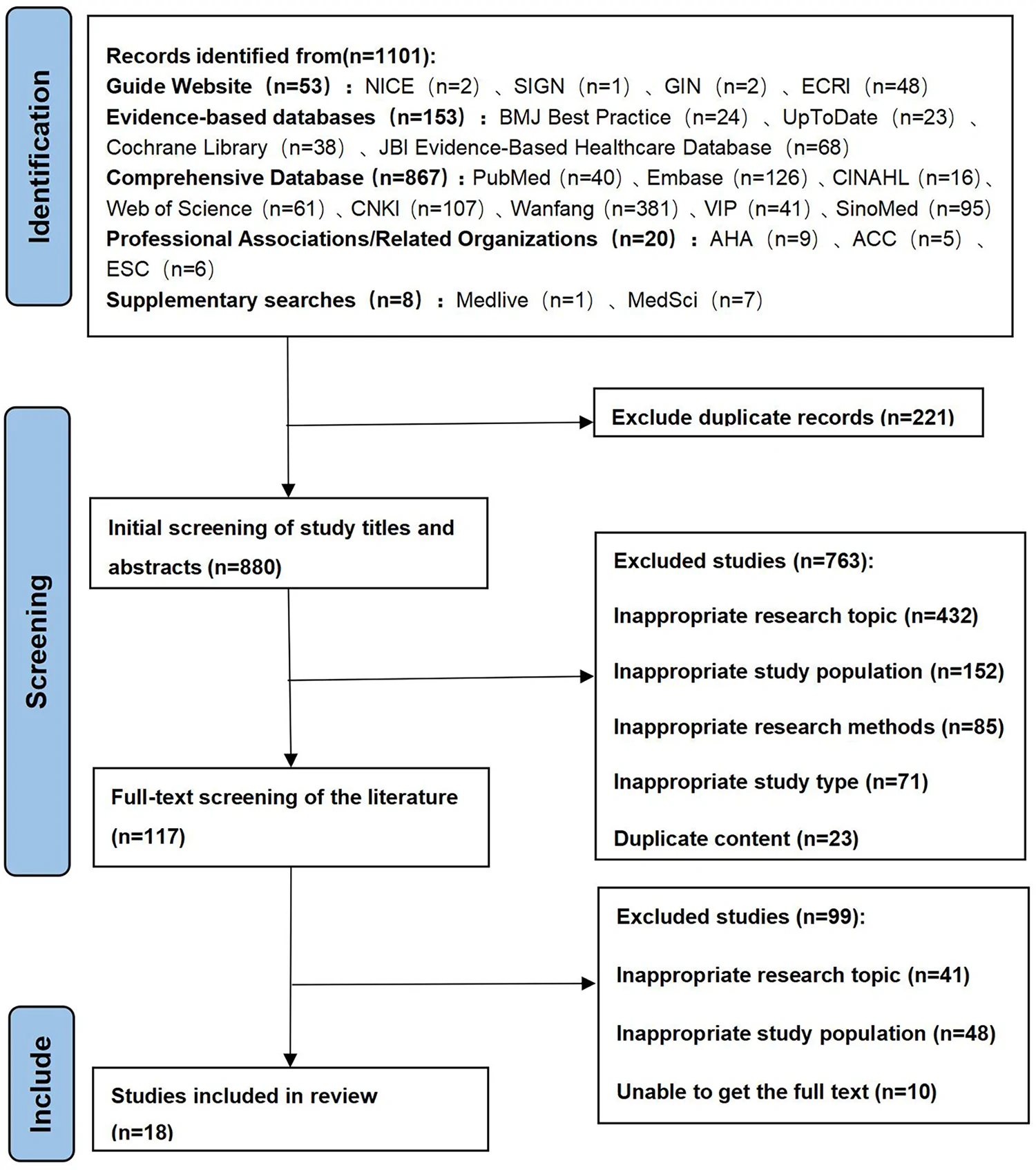
Literature screening process.

**Table 2:** Basic characteristics of included literature (*n* = 18).

Included literature	Year	Retrieval Source	Evidence Type	Topic/Focus
Rao et al. (16)	2025	AHA	Guideline	Management of the Acute Phase of Acute Coronary Syndrome
Vrints et al. (17)	2024	ESC	Guideline	Diagnosis and Treatment of Chronic Coronary Syndrome
Virani et al. (18)	2023	ACC	Guideline	Long-Term Comprehensive Management and Secondary Prevention of Chronic Coronary Artery Disease
Byrne et al. (19)	2023	ESC	Guideline	Comprehensive Management of Acute Coronary Syndrome
Interventional Cardiology Working Group, CSC (20)	2026	CINAHL	Expert consensus	Secondary Prevention and Long-Term Management Following PCI
Bueno et al. (21)	2025	ESC	Expert consensus	Integrated Management of Cardiovascular Disease and Mental Health
Goldfarb et al. (7)	2022	AHA	Expert consensus	Family Involvement in Cardiovascular Disease Management
Thomas et al. (22)	2019	AHA	Expert consensus	Home-Based Cardiac Rehabilitation Management
Beheshtaeen et al. (23)	2026	BMJ Best Practice	Systematic review	The Burden on Informal Caregivers of Heart Disease Patients and Its Determinants
Knowles et al. (24)	2021	Embase	Systematic review	The Effectiveness of Support Interventions for Caregivers of Heart Disease Patients
Chen et al. (25)	2025	MedSci	Evidence Summary	Summary of the Best Evidence for Discharge Planning for PCI Patients
Yang et al. (26)	2025	PubMed	RCT	Post-PCI Couple-Based Collaborative Management Intervention
Shen et al. (27)	2025	CINAHL	RCT	Family-Based Early Warning and Symptom Monitoring
Zheng et al. (28)	2024	UpToDate	RCT	Post-PCI Home-Based Telerehabilitation for the Heart
Zhang et al. (29)	2024	PubMed	Quasi-experimental study	The Effects of a Couple-Based Exercise Intervention on Exercise Adherence and Cardiopulmonary Function in Patients After PCI
Li et al. (30)	2024	CINAHL	Quasi-experimental study	Application of the Dyadic-Determinant Model of Disease Management in Home-Based Cardiac Rehabilitation Following PCI in the Elderly
Zhang et al. (31)	2021	CINAHL	Quasi-experimental study	A Trans-Theoretical Model of Family Empowerment: Its Impact on Health Behaviors and Caregiver Perceptions Among Young and Middle-Aged Patients Following PCI
Ma et al. (32)	2020	CINAHL	Quasi-experimental study	The Impact of Spousal Participation in Rehabilitation Exercises on Health Outcomes in Patients Following Stent Implantation

AHA stands for the American Heart Association, ACC stands for the American College of Cardiology, and ESC stands for the European Society of Cardiology.

### 3.2 Results of the quality assessment of the included evidence sources

#### 3.2.1 Results of the quality assessment of the guidelines

A total of 4 guidelines were included (16–19); see Table 3 for the results of the quality assessment.

**Table 3:** Results of quality evaluation of included guidelines.

Included literature	Standardized domain score (%)						Domains ≥60% (n)	Domains ≥30% (n)	Overall Appraisal Classification
	Scope and Purpose	Stakeholder involvement	Rigor of development	Clarity of presentation	Applicability	Editorial independence			
Rao et al. (16)	80.56%	69.44%	71.88%	80.56%	60.42%	83.33%	6	6	A
Vrints et al. (17)	80.56%	83.33%	73.96%	94.44%	62.50%	91.67%	6	6	A
Virani et al. (18)	75.00%	66.67%	62.50%	80.56%	64.58%	83.33%	6	6	A
Byrne et al. (19)	80.56%	72.22%	63.54%	83.33%	54.17%	75.00%	5	6	B

Standardized domain score = (actual score − minimum possible score)/(maximum possible score − minimum possible score) × 100%. For descriptive classification in this evidence summary, guidelines were categorized as Grade A when all six domain scores were ≥60%; Grade B when at least three domain scores were ≥30% but one or more domains were <60%; and Grade C when at least three domain scores were <30%.

#### 3.2.2 Results of the quality assessment of expert consensus

Of the four expert consensus documents included (7, 20–22), three (7, 21, 22) received a rating of “Yes” for all items; one (20) received a rating of “Unclear” for item 6, while all other items were rated “Yes.” The overall quality of the literature was high, and all documents were included.

#### 3.2.3 Quality assessment results of systematic reviews

A total of 2 systematic reviews were included (23, 24). For both reviews, all criteria were rated as “Yes” except for the item “Did the review assess the possibility of publication bias?”, which was rated as “No.” As the overall quality was high, both were included.

#### 3.2.4 Results of the quality assessment of the evidence summary

One evidence summary was included (25). With the exception of item 6, “Are there any inconsistencies with previous literature?”, which was rated as “Unclear,” all other items were rated as “Yes.” The overall quality was high, and the evidence summary was retained for inclusion.

#### 3.2.5 Results of the quality assessment of randomized controlled trials

Among the three included randomized controlled trials (26–28), the study by YANG et al. (26) rated items 2 and 6 as “Unclear” and items 4, 5, and 9 as “No”; the study by Shen Yan et al. (27) rated items 2, 4, and 6 as “Unclear” and item 5 as “No”; ZHENG et al. (28) rated items 2, 6, 8, and 9 as “Unclear,” items 4 and 5 as “No,” and all other items as “Yes.” Since all three studies involved nursing or behavioral interventions, implementing blinding posed certain difficulties; consequently, lower scores on relevant items are a common limitation of such studies. Although several methodological limitations were identified, particularly in relation to blinding, all three RCTs were retained for subsequent evidence synthesis following critical appraisal see Table 4 for details.

**Table 4:** Results of quality evaluation of included randomized controlled trials.

Included literature	1	2	3	4	5	6	7	8	9	10	11	12	13
Yang et al. (26)	Y	U	Y	N	N	U	Y	Y	N	Y	Y	Y	Y
Shen et al. (27)	Y	U	Y	U	N	U	Y	Y	Y	Y	Y	Y	Y
Zheng et al. (28)	Y	U	Y	N	N	U	Y	U	U	Y	Y	Y	Y

Items 1–13 correspond to the criteria of the JBI Critical Appraisal Checklist for Randomized Controlled Trials. Y = Yes; N = No; U = Unclear.

#### 3.2.6 Results of the quality assessment of quasi-experimental studies

Of the four quasi-experimental studies included (29–32), two studies (29, 32) received a rating of “Unclear” for Item 6, while the remaining two studies (30, 31) received a rating of “No” for Item 6; all other items were rated “Yes.” Despite the identified methodological limitations, all four quasi-experimental studies were retained for subsequent evidence synthesis following critical appraisal. The detailed appraisal results are presented in Table 5.

**Table 5:** Results of quality evaluation of included quasi-experimental studies.

Included literature	1	2	3	4	5	6	7	8	9
Zhang et al. (29)	Y	Y	Y	Y	Y	U	Y	Y	Y
Li et al. (30)	Y	Y	Y	Y	Y	N	Y	Y	Y
Zhang et al. (31)	Y	Y	Y	Y	Y	N	Y	Y	Y
Ma et al. (32)	Y	Y	Y	Y	Y	U	Y	Y	Y

Items 1–9 correspond to the criteria of the JBI Critical Appraisal Checklist for Quasi-Experimental Studies. Y = Yes; N = No; U = Unclear.

### 3.3 Summary of synthesized evidence

Following evidence synthesis, 27 evidence-based recommendations were identified across five domains: assessment and planning, education and decision-making, self-management and rehabilitation, psychosocial support, and continuity of care. The synthesized evidence supports identifying and engaging the caregiver selected by the patient, with the patient's consent, and assessing both patient and caregiver needs, psychological status, caregiver burden, and readiness for collaboration. Recommended management strategies include joint education and shared decision-making; caregiver-supported medication adherence, lifestyle modification, exercise rehabilitation, symptom monitoring, and emergency-response skills; and psychosocial assessment, support, and referral for both members of the dyad. Continuity-of-care recommendations emphasize coordinated discharge preparation, early structured patient–caregiver follow-up, technology-enabled support, and ongoing monitoring of cardiovascular risk factors, adherence, and psychological needs. Detailed supporting evidence, evidence levels, and recommendation grades are provided in Table 6.

**Table 6:** Summary of the best evidence for dyadic disease management following coronary stent implantation.

Topic	Evidence-based recommendation	Evidence level	Recommendation grade
Assessment and planning	1. Caregiver identification and engagement: Identify the family caregiver whom the patient wishes to involve in care and, with the patient's consent, engage that caregiver as a partner in ongoing post-stenting disease management (7).	5b	B
	2. Psychological assessment and screening: Assess psychological well-being in post-stenting patients using validated tools; the Whooley Questions or PHQ-2 may be used for depression screening and the GAD-2 for anxiety screening, with positive results prompting further assessment or referral (18, 21). For patients undergoing stenting in the context of ACS, psychological assessment before discharge should be considered (19).	5b	B
	3. Caregiver capacity and burden assessment: Assess caregivers’ caregiving capacity, burden, and support needs (7, 23); caregiver burden may be quantified using a validated instrument, such as the Zarit Burden Interview (ZBI) (23). Family and available support resources should also be considered when planning caregiver support (23, 25).	1a	B
	4. Caregiver psychological assessment: Routinely assess caregivers for anxiety, depression, and psychological distress (7, 21, 23, 24); validated instruments such as the Hospital Anxiety and Depression Scale (HADS) may be used to quantify anxiety and depressive symptoms (24).	5b	B
	5. Readiness for dyadic collaboration: Assess the patient's and caregiver's willingness to participate in shared management, understanding of management tasks and goals, and expected roles (26, 30–32). Where expectations differ, facilitate discussion and role clarification; family involvement and information sharing should remain consistent with the patient's preferences and consent (7, 26).	1c	B
	6. Collaborative goal setting: Guide patients and caregivers to jointly establish individualized goals for medication adherence, risk-factor and lifestyle management, exercise rehabilitation, symptom monitoring, and follow-up (26, 30–32). A decision-balance approach and SMART principles may be used to translate these goals into a specific collaborative management plan and clarify each partner's responsibilities (26).	1c	B
Education and decision-making	7. Structured joint education: Provide structured joint education to patients and caregivers (7, 26) covering disease and complication knowledge, medication management, risk-factor and lifestyle management, diet and exercise rehabilitation, symptom recognition, and follow-up (18, 20, 25, 26). Use the teach-back method to verify understanding and correct misunderstandings as needed (26).	1c	A
	8. Stage-tailored empowerment education: Tailor rehabilitation education and caregiver support to the patient's stage of behavior change and readiness for rehabilitation, and progressively adjust educational content and support as readiness changes (31).	2c	B
	9. Caregiver-specific skills training: Provide caregivers with targeted training based on identified knowledge and skill gaps, focusing on medication support, recognition of warning symptoms and appropriate responses, and communication/support skills (7, 24, 26).	1a	A
	10. Joint risk-factor management education: Educate patients and caregivers on practical risk-factor management, including home blood pressure and heart rate monitoring, lipid management, heart-healthy eating, and recognition of orthostatic hypotension (26, 27, 30).	1c	B
	11. Shared decision-making with caregiver involvement: Engage patients in shared decision-making and incorporate their values, preferences, and goals into treatment decisions (16–18). When the patient wishes, and with the patient's consent, involve the identified caregiver in relevant discussions and information sharing (7).	5b	B
Self-management and rehabilitation	12. Caregiver-supported medication management: Educate patients and caregivers on the prescribed medication regimen and the importance of sustained adherence (16, 18, 20, 26). With the patient's consent, caregivers should support medication-taking and help monitor for clinically relevant adverse effects, particularly bleeding during antithrombotic therapy (16, 18, 20, 26).	1c	A
	13. Family-supported diet and lifestyle management: Encourage a heart-healthy dietary pattern, such as a Mediterranean-style diet, rich in vegetables, fruits, and whole grains and low in saturated fat and sodium (17, 18, 20). Caregivers may support meal preparation, smoking cessation, alcohol reduction, and a smoke-free home environment (17, 18, 20, 26).	5b	A
	14. Family-supported exercise rehabilitation: Encourage ≥150 min/week of moderate-intensity aerobic activity, individualized to the patient's clinical status (17, 18, 22). During home-based rehabilitation, caregivers may support exercise (28, 29) and should be taught to recognize warning symptoms requiring exercise cessation, such as chest pain, dyspnea, or dizziness (22).	1c	A
	15. Couple-based exercise participation: Where feasible, encourage patients and spouses to exercise together within an individualized, progressive cardiac rehabilitation plan; a structured program of approximately 12 weeks, 3 sessions/week, at moderate intensity (Borg RPE 11–14) may be used as a practical reference to improve exercise adherence and promote mutual health benefits (29, 32).	2c	B
	16. Caregiver emergency-response training: Train caregivers to recognize warning signs such as chest pain, dyspnea, and altered consciousness and respond promptly by activating emergency medical services and following the patient's prescribed emergency plan (20, 27).	1c	A
	17. Triadic collaborative management: Establish patient–caregiver–provider collaboration to jointly develop management plans, provide ongoing feedback and emotional support, and monitor health behaviors (26, 30).	1c	B
	18. Caregiver role recognition and engagement: Recognize caregivers as active partners in care and, with the patient's consent, involve them in relevant care planning and rehabilitation; clinicians should value caregivers’ observations and feedback and provide support appropriate to their caregiving role (7, 24).	5b	B
Psychosocial support	19. Family-supported psychological care: For patients with anxiety or depression, encourage caregivers to provide supportive communication, including active listening and empathic responses (7), and support participation in appropriate non-pharmacological psychological interventions or professional referral (21).	5b	B
	20. Family presence during resuscitation and procedures: When clinically feasible and consistent with patient preferences, offer family members the option to be present during resuscitation or invasive procedures, provided that patient care and safety are not compromised; family presence may reduce psychological distress (7).	5b	B
	21. Stage-tailored caregiver psychosocial support: Tailor caregiver support to the phase of care and identified needs: during the acute and early recovery phase, prioritize psychological support, crisis support, and timely referral when indicated; during long-term management, emphasize role adaptation, stress management, coping skills, and sustained support (7, 21, 23, 24).	1b	B
	22. Caregiver support and referral: Connect caregivers with peer support, community resources, and professional psychological services according to identified needs, and ensure access to ongoing support throughout post-stenting care (7, 21, 23, 24).	1a	B
Continuity of care	23. Discharge transition coordination: Within approximately 24 h before discharge, verify discharge readiness and, with the patient's consent, confirm discharge destination, transportation, medication, and support-resource arrangements with the caregiver (7, 25); designate a care coordinator to oversee implementation and information handover (25).	2a	A
	24. Technology-enabled remote follow-up: Use telephone or digital platforms for post-discharge remote monitoring, health education, adherence support, and rehabilitation guidance; where appropriate, wearable devices may support home-based rehabilitation (22, 25, 28). With the patient's consent, caregivers may participate in remote follow-up and collaborative management (7, 26).	1c	B
	25. Early post-discharge dyadic follow-up: Conduct structured patient–caregiver follow-up early after discharge, with contacts at approximately 1, 2, and 4 weeks, to assess adherence, goal attainment, and patient–caregiver collaboration (25–27); maintain closer surveillance during the second to third months, when adherence may decline (26, 27).	1c	B
	26. Long-term follow-up and risk-factor monitoring: Conduct follow-up at least annually to reassess cardiovascular risk factors, medication adherence, and lifestyle behaviors (17, 18, 20). Recheck the lipid profile 4–8 weeks after initiation or adjustment of lipid-lowering therapy (16), and encourage caregiver participation in ongoing follow-up, consistent with patient preferences (7, 26).	5b	A
	27. Stepped psychological screening and care: Conduct psychological screening after acute cardiovascular events, during routine follow-up, and when clinically indicated (18, 19, 21); provide further assessment, intervention, or referral according to symptom severity (21). With the patient's consent, involve caregivers in psychological support and follow-up as appropriate (7, 21, 24).	5b	A

The Whooley Questions refer to the Whooley Depression Screening Questions; PHQ-2 refers to the Patient Health Questionnaire-2; GAD-2 refers to the Generalized Anxiety Disorder-2; ZBI refers to the Zarit Burden Interview; HADS refers to the Hospital Anxiety and Depression Scale; SMART refers to the goal-setting principles of Specific, Measurable, Achievable, Relevant, and Time-bound.

## 4 Discussion

The five evidence domains identified in this review are interrelated rather than independent components of post-stenting care. Together, they form a continuous and adaptive dyadic management pathway in which assessment informs education and collaborative planning, self-management and rehabilitation translate shared goals into daily behaviors, psychosocial support sustains the participation of both members of the dyad, and continuity of care enables ongoing reassessment and adjustment.

### 4.1 Assessment and planning: matching dyadic involvement to needs and capacity

Effective dyadic management begins with identifying an appropriate caregiver rather than assuming that any available family member should participate. The family caregiver whom the patient wishes to involve should be identified and, with the patient's consent, engaged as a partner in ongoing post-stenting disease management (7). This patient-directed approach is important because caregiver participation should support patient self-management without replacing patient autonomy. Accordingly, the form and extent of caregiver involvement should reflect the patient's preferences together with the caregiver's willingness and capacity to participate.

Assessment should subsequently extend to both members of the dyad. For patients, psychological well-being should be assessed using validated tools; the Whooley Questions or Patient Health Questionnaire-2 (PHQ-2) may be used for depression screening and the Generalized Anxiety Disorder-2 (GAD-2) for anxiety screening, with positive findings prompting further assessment or referral (18, 21). For patients undergoing coronary stenting in the context of acute coronary syndrome, psychological assessment before discharge should also be considered (19). For caregivers, assessment should address caregiving capacity, perceived burden, psychological distress, and available support resources (7, 21, 23–25). The Zarit Burden Interview (ZBI) is a validated instrument for assessing perceived caregiver burden, whereas the Hospital Anxiety and Depression Scale (HADS) can be used to quantify symptoms of anxiety and depression (23, 24). These assessments have practical implications because substantial caregiver burden or psychological distress may reduce the capacity to provide sustained support.

Assessment should also determine readiness for collaboration. Patients and caregivers may differ in their understanding of management goals, expected responsibilities, or desired degree of family involvement. Evidence therefore supports assessing willingness to participate, understanding of management tasks and goals, and expected roles, followed by discussion and role clarification when expectations differ (7, 26, 30–32). On this basis, clinicians can guide patients and caregivers to establish individualized goals for medication adherence, risk-factor and lifestyle management, exercise rehabilitation, symptom monitoring, and follow-up; SMART principles may be used to translate these goals into specific and measurable collaborative actions (26, 30–32).

Thus, the purpose of dyadic assessment is not to maximize caregiver involvement, but to determine a form and intensity of participation that are appropriate and sustainable for each patient–caregiver dyad. A caregiver with adequate knowledge, willingness, and psychological capacity may assume a more active collaborative role, whereas a caregiver experiencing substantial burden may require additional support rather than additional responsibilities. This needs- and capacity-based approach provides the foundation for subsequent education, behavioral management, and continuity of care.

### 4.2 Education and decision-making: aligning knowledge, roles, and goals

Once the needs and readiness of the dyad have been identified, structured joint education becomes an important means of establishing a shared understanding of post-stenting management. Education should include both patients and caregivers and address disease and complication knowledge, medication management, risk-factor and lifestyle management, diet, exercise rehabilitation, symptom recognition, and follow-up (7, 18, 20, 25, 26). The teach-back method can be used to verify understanding and correct misconceptions rather than assuming that information delivery alone has resulted in adequate comprehension (26).

Education should also be tailored to rehabilitation readiness and identified skill gaps. Rehabilitation education and caregiver support can be progressively adjusted according to the patient's stage of behavioral change and readiness for rehabilitation (31). Caregivers with identified knowledge or skill deficits should receive targeted training in medication support, recognition of warning symptoms and appropriate responses, and communication and supportive skills (7, 24, 26). Practical risk-factor education should also prepare patients and caregivers for home blood pressure and heart-rate monitoring, lipid management, heart-healthy eating, and recognition of orthostatic hypotension (26, 27, 30).

Shared decision-making further connects education with subsequent management behavior. Treatment decisions should remain centered on the patient's values, preferences, and goals (16–18), while the identified caregiver may participate in relevant discussions and information sharing when the patient wishes and provides consent (7). Joint education and shared decision-making therefore perform complementary functions: education establishes a common knowledge base, whereas shared decision-making aligns expectations, roles, and responsibilities. Together, they provide the cognitive and relational foundation for subsequent collaborative self-management.

### 4.3 Self-management and rehabilitation: translating collaboration Into sustained secondary prevention

The practical value of dyadic management depends on whether shared understanding and agreed goals can be translated into sustained daily behaviors. Medication adherence, lifestyle modification, exercise rehabilitation, symptom monitoring, and emergency preparedness represent key areas in which caregiver support can supplement, rather than replace, patient self-management.

Medication management is one of the most direct forms of collaboration. Patients and caregivers should understand the prescribed medication regimen and the importance of sustained adherence; with the patient's consent, caregivers may support medication-taking and help monitor clinically relevant adverse effects, particularly bleeding during antithrombotic therapy (16, 18, 20, 26). Family participation may similarly facilitate lifestyle modification. Heart-healthy dietary patterns are recommended, and caregivers may support meal preparation, smoking cessation, alcohol reduction, and maintenance of a smoke-free home environment (17, 18, 20, 26). In this way, selected secondary-prevention behaviors can be reinforced through everyday family routines without shifting overall responsibility for disease management away from the patient.

Exercise rehabilitation provides another important opportunity for collaboration. Individualized aerobic exercise, generally aiming for at least 150 min/week of moderate-intensity activity when clinically appropriate, is supported by cardiovascular recommendations (17, 18, 22). During home-based rehabilitation, caregivers may support exercise participation and should be taught to recognize warning symptoms requiring exercise cessation, such as chest pain, dyspnea, or dizziness (22, 28, 29). Where feasible, spouse-involved exercise may provide additional support for adherence. A structured program of approximately 12 weeks, three sessions per week, at moderate intensity corresponding to a Borg rating of perceived exertion of 11–14 may be used as a practical reference (29, 32). Because this specific exercise format is derived from context-dependent intervention studies, it should be considered an adaptable option rather than a universal prescription and should be individualized according to clinical status, rehabilitation capacity, and family circumstances.

Dyadic self-management should also include preparation for clinical deterioration. Caregivers should be trained to recognize warning signs such as chest pain, dyspnea, and altered consciousness and to activate emergency medical services according to the patient's prescribed emergency plan (20, 27). More broadly, patient–caregiver–provider collaboration can support joint management planning, behavioral monitoring, ongoing feedback, and problem solving (26, 30). Clinicians should also recognize caregivers' observations and provide support appropriate to their caregiving role (7, 24).

Overall, these findings highlight an important distinction between the clinical targets of secondary prevention and the way in which those targets are implemented. Medication adherence, lifestyle modification, risk-factor control, and exercise remain patient-centered clinical goals, whereas dyadic management provides an additional mechanism for sustaining these behaviors in the home environment. The intensity and form of caregiver participation should therefore be adapted to patient preferences, caregiver capacity, and clinical context.

### 4.4 Psychosocial support: sustaining the capacity of both members of the dyad

Psychological health is closely related to the functioning of the patient–caregiver dyad. Following PCI, patients may experience anxiety, depression, or concerns regarding recurrent cardiovascular events, while caregivers may simultaneously experience distress and burden associated with long-term caregiving responsibilities (21, 23, 24, 34). Psychological difficulties in either member may affect communication, adherence, rehabilitation participation, and the family's capacity to sustain disease-management activities.

For patients with anxiety or depressive symptoms, caregivers may be encouraged to provide supportive communication through active listening and empathic responses and to support participation in appropriate non-pharmacological psychological interventions or professional referral (7, 21). Caregiver well-being, however, requires independent attention rather than being considered only in relation to the support provided to the patient. Support should be matched to the phase of care and identified needs: during the acute and early recovery phase, psychological support, crisis support, and timely referral may be prioritized; during long-term management, greater emphasis may be placed on role adaptation, stress management, coping skills, and sustained support (7, 21, 23, 24). When needs exceed available family or routine clinical resources, caregivers should be connected with peer support, community resources, or professional psychological services (7, 21, 23, 24).

When clinically feasible, consistent with patient preferences, and without compromising patient care or safety, family members may also be offered the option to be present during resuscitation or invasive procedures (7). Because this practice is highly dependent on clinical circumstances and patient preference, it should be considered a context-sensitive option rather than a routine requirement.

The synthesized evidence therefore indicates a reciprocal relationship within dyadic management: caregivers can contribute to patients' psychological support, but their own psychological well-being influences whether they can sustain that supportive role. Consequently, assessment of caregiver burden and psychological distress should be linked to subsequent support and referral rather than treated as isolated screening activities. Psychosocial support therefore functions not only as an outcome-oriented intervention but also as a mechanism for sustaining long-term participation in secondary prevention and rehabilitation.

### 4.5 Continuity of care: maintaining a longitudinal feedback loop From hospital to home

The preceding components are unlikely to remain effective if they are confined to hospitalization. Transition to the home environment changes how medication management, rehabilitation, symptom monitoring, and psychosocial support are delivered; continuity of care is therefore required to reassess progress and adapt the management plan over time. Observational evidence has also examined transitional care as an approach to supporting self-management among older patients after PCI, further highlighting the importance of extending disease management beyond hospital discharge (33).

Discharge transition should begin before the patient leaves hospital. Within approximately 24 h before discharge, discharge readiness should be verified and, with the patient's consent, clinicians should confirm the discharge destination, transportation, medication arrangements, and available support resources with the caregiver; a designated care coordinator can support information handover and implementation (7, 25). After discharge, telephone or digital platforms may be used for remote monitoring, health education, adherence support, and rehabilitation guidance, while wearable devices may support home-based rehabilitation where appropriate (22, 25, 28). With patient consent, caregivers may participate in remote follow-up and collaborative management (7, 26).

The evidence also provides a practical temporal structure for follow-up. Structured patient–caregiver contacts at approximately 1, 2, and 4 weeks after discharge can be used to assess adherence, goal attainment, and patient–caregiver collaboration, with closer surveillance during the second and third months when adherence may decline (25–27). Long-term follow-up should reassess cardiovascular risk factors, medication adherence, and lifestyle behaviors at least annually (17, 18, 20). For patients in whom lipid-lowering therapy is initiated or adjusted, the lipid profile should be reassessed 4–8 weeks after the treatment change (16). Psychological screening should also be repeated after acute cardiovascular events, during routine follow-up, and when clinically indicated, followed by further assessment, intervention, or referral according to symptom severity (18, 19, 21). Caregiver participation in psychological support and follow-up should remain consistent with patient consent and identified needs (7, 21, 24).

Continuity of care therefore functions as the feedback mechanism connecting the five evidence domains. Initial assessment identifies needs and capacity; education and shared decision-making establish aligned goals; self-management and rehabilitation translate these goals into daily behaviors; psychosocial support helps preserve the ability of both members to participate; and follow-up identifies changes in adherence, caregiver capacity, psychological status, or cardiovascular risk that require modification of the management plan.

From a clinical perspective, the synthesized evidence supports a stepwise and individualized rather than uniform approach to caregiver involvement. Core practice should include patient-authorized caregiver engagement, assessment of both members of the dyad, joint education and collaborative goal setting, caregiver-supported secondary-prevention behaviors, psychosocial support, and structured continuity of care. More context-dependent strategies—including specific couple-based exercise programs, family presence during invasive procedures, wearable-device-supported rehabilitation, and the intensity of caregiver participation—should be individualized according to patient preferences, clinical status, caregiver capacity, and available healthcare resources. This distinction between core management functions and adaptable implementation strategies may facilitate translation of the evidence across hospital, community, and home settings.

### 4.6 Limitations

The breadth of evidence required to address post-stenting dyadic disease management was characterized by variation in study design, population characteristics, intervention content, and modes of caregiver participation. To support methodological rigor, design-specific appraisal tools were applied, and recommendations were evaluated according to evidence level and recommendation grade. Some primary studies were conducted in single-center settings with relatively small samples, and direct evidence remains limited for certain caregiver-involvement strategies, with some recommendations also drawing on secondary evidence or expert guidance. Eligibility was restricted to Chinese- and English-language publications, and residual publication bias cannot be completely excluded. Future multicenter and implementation studies may further evaluate the applicability and effectiveness of the integrated dyadic management pathway.

## 5 Conclusions

This evidence summary integrates previously dispersed evidence on post-stenting patient–caregiver management into a structured and continuous dyadic disease-management pathway. Effective implementation should begin with patient-authorized caregiver engagement and assessment of both members of the dyad, followed by joint education and collaborative goal setting, caregiver-supported secondary prevention and rehabilitation, psychosocial support, and structured continuity of care.

The framework does not imply uniform or maximal caregiver involvement. Rather, the form and intensity of participation should be adapted to patient preferences, caregiver capacity, clinical status, and available healthcare resources. By linking patient self-management with appropriately supported caregiver participation across the transition from hospital to home and long-term follow-up, the synthesized recommendations provide a practice-oriented evidence base for incorporating patient–caregiver collaboration into long-term disease management after coronary stent implantation.
